# Curvilinear relationship between life's crucial 9 and metabolic syndrome in U.S. adults: a cross-sectional study

**DOI:** 10.3389/fendo.2025.1559413

**Published:** 2025-04-15

**Authors:** Bo Wang, Chunqi Jiang, Pingping Yu, Zhen Nie, Ning Wang, Xin Zhang

**Affiliations:** ^1^ Central Hospital of Jinan City, Jinan, Shandong, China; ^2^ Affiliated Hospital of Shandong University of Traditional Chinese Medicine, Jinan, Shandong, China; ^3^ Wendeng District People’s Hospital of Weihai City, Wendeng District, weihai, Shandong, China; ^4^ Shandong University of Traditional Chinese Medicine, Jinan, Shandong, China

**Keywords:** life’s crucial 9, metabolic syndrome, curvilinear, NHANES, cross-sectional study

## Abstract

**Background:**

Metabolic Syndrome (MetS) is closely linked to cardiovascular disease. However, no studies have examined the relationship between Life’s Crucial 9 (LC9) and MetS. Our goal is to investigate the potential association between LC9 and MetS.

**Methods:**

We employed a weighted multivariate logistic regression model to evaluate the relationship between LC9, health behavior score, health factors score, and MetS. To assess the robustness of this association, we conducted sensitivity analyses. Furthermore, we utilized smooth curve fitting to investigate the potential curvilinear relationships between LC9, health behavior score, health factors score, and MetS. To pinpoint inflection points, we integrated recursive partitioning algorithms with a two-stage linear regression model. Additionally, we performed stratified analyses to explore heterogeneity across different population subgroups.

**Results:**

Our study included a total of 28,555 participants. In the regression model that accounted for all covariates, the OR for LC9 and MetS was 0.941 (0.939, 0.944), indicating a significant negative correlation between the two. Smooth curve analysis confirmed a curvilinear relationship between LC9 and MetS, with an inflection point at 70.56. The negative correlation was evident both before and after the inflection point, with a more pronounced effect after the inflection point. Subgroup analyses of Health behavior score and Health factors score, as well as stratified analyses by age, sex, and BMI, showed that all groups exhibited curvilinear relationships consistent with the overall pattern.

**Conclusion:**

The curvilinear relationship between LC9 scores and metabolic syndrome indicates that higher LC9 scores act as a protective factor against MetS.

## 1 Introduction

Metabolic Syndrome (MetS) is characterized by a cluster of metabolic disorders, including elevated fasting blood glucose, high blood pressure, abdominal obesity, increased triglycerides (TG), and decreased high-density lipoprotein cholesterol (HDL-C) (1). It is marked by high prevalence and significant potential harm. It is estimated that 1.5 billion people worldwide develop MetS each year (2). Approximately one-third of the U.S. population is affected by MetS (3). Research indicates that MetS is closely linked to an increased risk of cardiovascular disease (CVD), non-alcoholic fatty liver disease, and diabetes (4, 5). Moreover, MetS is significantly associated with increased mortality, particularly from cardiovascular disease (6). However, there are currently no targeted drugs for MetS (7). Therefore, timely identification and intervention are crucial to prevent and delay the onset and progression of MetS.

Life’s Crucial 9 (LC9) is an extension of Life’s Essential 8 (LE8) introduced by the American Heart Association, incorporating mental health into the framework. LC9 is a comprehensive and actionable metric that integrates four health behaviors, four health factors, and mental health to improve cardiovascular health (8). Research has shown that a high LE8 score is significantly associated with a reduced risk of CVD and lower mortality rates (9). Furthermore, a higher LE8 score is linked to lower all-cause and disease-specific mortality in individuals with chronic kidney disease and cancer survivors (10, 11). Moreover, depression is strongly linked to adverse health conditions. Depression is an independent risk factor for adverse outcomes in coronary heart disease and may contribute to vascular aging (12). It is also associated with conditions such as dyslipidemia and visceral adiposity (13, 14).

Previous studies have shown that the LE8 score is negatively correlated with all-cause mortality and CVD mortality in individuals with MetS, indicating that a higher LE8 score is a protective factor against MetS (15). Additionally, major depressive disorder is closely linked to blood glucose and lipid levels, including fasting blood glucose, HDL-C, and total cholesterol (TC) (16). However, the association between LC9 scores and MetS remains unexplored. Our goal is to utilize data from the U.S. NHANES to examine the potential relationship between LC9 scores and MetS, providing evidence for the prevention and management of MetS.

## 2 Materials and methods

### 2.1 Study design and participants

**Figure 1:**
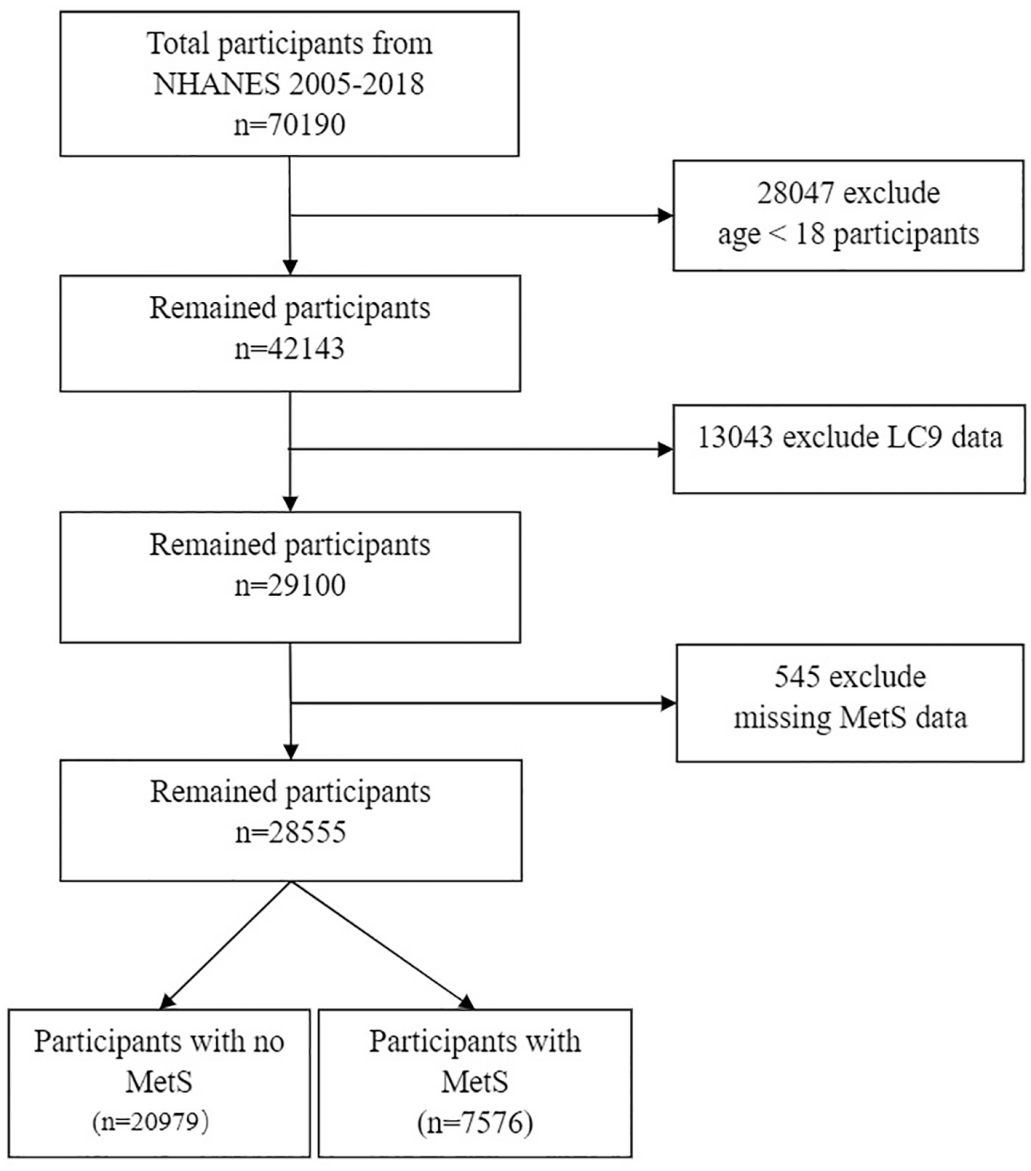
Flow chart of sample selection from the 2005-2018. MetS, Metabolic syndrome; LC9, Life’s Crucial 9.

Our research harnessed data from the United States’ NHANES, spanning a decade from 2005 to 2018 across eight survey cycles. The NHANES database is an invaluable trove of information, encompassing demographic details, lifestyle factors, self-reported health metrics, and comprehensive blood biochemical assessments. Data collection is meticulously conducted through household interviews, mobile examination centers, and rigorous laboratory tests. This resource is generously accessible to the research community without the need for specific permissions. The research protocol was meticulously approved by the National Center for Health Statistics Institutional Review Board, with all participants providing their informed consent in writing. To safeguard privacy, all personal identifiers were meticulously anonymized. During the data preparation phase of our study, we prudently excluded participants under the age of 18, totaling 28047 individuals. Due to the presence of incomplete data regarding the Healthy Eating Index-2015 (HEI-2015), physical activity, smoking exposure, sleep duration, Body Mass Index (BMI), blood lipids, blood sugar, blood pressure, and Patient Health Questionnaire-9 (PHQ-9), we had to exclude an additional 13043 individuals. An additional 545 participants were excluded due to the absence of MetS data. Our study ultimately encompassed a total of 28,555 participants, with 20,979 individuals not diagnosed with MetS and 7,576 individuals diagnosed with MetS, as illustrated in **Figure 1**.

### 2.2 Study variables

#### 2.2.1 Definition of LC9

The LC9 score is calculated by averaging two components: the LE8 score and the PHQ-9 score (17). The LE8 score, introduced by the AHA in 2022, is detailed in Table LE8 and is based on eight metrics of cardiovascular health (18). These metrics cover four health behaviors—diet, physical activity, nicotine exposure, and sleep—and four health factors—BMI, non-HDL cholesterol, blood glucose, and blood pressure. Each metric is scored from 0 to 100, and the LE8 score is the mean of these values. The PHQ-9 score categorizes depression into five levels (100, 75, 50, 25, and 0) corresponding to the PHQ-9 scale ranges of 0-4, 5-9, 10-14, 15-19, and 20-27, respectively (19). Dietary scores are determined using the HEI-2015 quintiles (20), which are calculated from two 24-hour dietary recalls and the USDA’s Food Pattern Equivalents data. Information on physical activity, nicotine exposure, sleep duration, and diabetes history is collected through standardized questionnaires. During the physical examination, height, weight, Waist circumference and blood pressure are measured, and BMI is calculated. Blood samples are taken for laboratory analysis to measure lipid profiles, fasting blood glucose, and HbA1c levels.

#### 2.2.2 Definition of metabolic syndrome

In this study, MetS was evaluated using the criteria established by the National Cholesterol Education Program’s Adult Treatment Panel III in the United States (21), which have been extensively validated through extensive epidemiological research. Participants were diagnosed with MetS if they met three or more of the following criteria: 1) Hypertriglyceridemia: Serum triglycerides ≥150 mg/dL (1.7 mmol/L) or on lipid-lowering medication; 2) Central obesity: Waist circumference ≥102 cm for men and ≥88 cm for women; 3) Reduced HDL-C: Serum HDL-C levels < 40 mg/dL (1.03 mmol/L) for men and < 50 mg/dL (1.29 mmol/L) for women; 4) Hypertension: Systolic blood pressure ≥130 mmHg or diastolic blood pressure ≥85 mmHg; 5) Hyperglycemia: Fasting plasma glucose ≥100 mg/dL (5.6 mmol/L) or on antidiabetic medication (22).

#### 2.2.3 Assessment of other variables

The covariates considered in our study were collected by the CDC using computer-assisted personal interviews and mobile examination centers. These include age, gender, race, education level, marital status, poverty-income ratio, smoking status, alcohol consumption, recreational activity, diabetes, hypertension, cardiovascular disease, Stroke, and HDL-C. Race was categorized as Mexican American, non-Hispanic white, non-Hispanic black, other Hispanic, or other race. Education levels were divided into less than high school, high school, and more than high school; marital status into never married, married/living with partner, divorced/widowed/separated; smoking status was divided into three distinct groups: ‘Never’ defined as less than 100 cigarettes in one’s lifetime; ‘Former’ for those with a history of smoking but have quit; and ‘Now’ designated for those who continue to smoke (23). Drinking status was divided into three categories: “Never” includes individuals who have consumed fewer than 12 drinks in their lifetime, “Former” encompasses those who have a history of alcohol consumption but no longer drink, and “Now” refers to those who continue to consume alcohol (15). Participation in recreational activities was binary, recorded as ‘Yes’ or ‘No’. Diabetes diagnosis, including pre-diabetes, was based on meeting at least one of the following criteria: 1. Fasting blood glucose above 7.0 mmol/L; 2. Hemoglobin A1c (HbA1c) 6.5% or higher; 3. Random blood glucose level of at least 11.1 mmol/L; 4. Blood glucose level of 11.1 mmol/L or higher after a 2-hour oral glucose tolerance test; 5. A formal diagnosis of diabetes by a healthcare provider; 6. Impaired fasting glucose ranging from 6.11 to 7.0 mmol/L or impaired glucose tolerance, oral glucose tolerance test levels between 7.7 and 11.1 mmol/L. Hypertension was determined by one or more of the following conditions: 1. Systolic blood pressure reading of 140 mmHg or higher;2. Diastolic blood pressure of 90 mmHg or higher; 3. Current use of antihypertensive medication; 4. Self-reported hypertension. Cardiovascular disease was determined through a medical history questionnaire, recording whether participants had been diagnosed with coronary artery disease, congestive heart failure, or had a history of heart attack (24). Stroke was evaluated using self-reported questionnaires by asking participants if they had been informed of a stroke by a doctor or health professional. The question was, “Has a doctor or health professional ever told you that you had a stroke?” If the participant responded “yes,” they were classified as having experienced a stroke (25). Peripheral blood samples were collected in the morning after fasting for at least eight hours, and HDL-C in serum was quantified using either direct immunoassay or precipitation techniques.

### 2.3 Statistical analysis

The study’s demographic representation was enhanced through appropriate weighting of research data. We managed missing data by imputation, utilizing predictive mean matching for continuous variables and logistic regression for binary variables. Participants were divided into those with and without MetS according to their initial characteristics. Mean (95% CIs) depicted continuous variables, while percentages described categorical variables. To examine the relationship between LC9 and MetS, we utilized weighted logistic regression analysis, presenting results as odds ratios (ORs) and their corresponding 95% confidence intervals (95% CI). Similarly, we explored the associations between Health behavior score, Health factors score, and MetS using this method. LC9 was categorized into quartiles, while Health behavior score and Health factors score were classified as Low (0–49), Moderate (50–79), and High (80–100). Sensitivity analyses were conducted to verify the stability of the observed correlations between LC9, Health behavior score, and Health factors score with MetS. We stratified LC9 into quartiles and performed sensitivity analyses to confirm the stability of the observed associations between LC9, and MetS. Subsequently, smooth curve fitting was employed to investigate the curvilinear relationships between LC9, Health behavior score, and Health factors score with MetS, utilizing generalized additive models to identify nonlinear trends. Once non-linearity was established, the inflection point was identified using a recursive algorithm, which then informed the creation of a two-piecewise linear regression model. Stratified analyses were performed to investigate the stability and variability in the relationships between LC9, Health behavior score, and Health factors score with MetS. Statistical analyses were carried out using R (version 4.2.0) and EmpowerStats software (http://www.empowerstats.com), with statistical significance set at a P-value of less than 0.05.

## 3 Results

### 3.1 Baseline characteristics

**Table 1** presents the demographic and clinical profiles of participants, distinguishing those with MetS from those without. Individuals with MetS are significantly older, with a mean age of 56.11 years (95% CI: 55.59-56.63) compared to 45.75 years (95% CI: 45.25-46.25) for those without MetS. They also exhibit a higher BMI of 33.69 kg/m², elevated fasting glucose levels at 122.43 mg/dl, increased HbA1c levels at 6.19%, and a lower LC9 score of 60.50. Additionally, their income-to-poverty ratio is lower, at 2.92 (95% CI: 2.84-3.00). Participants with MetS are more likely to be female (53.11%), divorced, widowed, or separated (24.14%), have lower educational attainment, and not participate in recreational activities (58.82%). They are also more likely to be former drinkers (32.81%), former smokers (32.81%), and have a higher prevalence of diabetes (60.47%), hypertension (72.34%), CVD (18.94%), and depression (10.26%).

**Table 1:** Baseline characteristics of participants.

	No MetS (n=20979)	With MetS (n=7576)	P-value
Age (year)	45.75 (45.25,46.25)	56.11 (55.59,56.63)	<0.0001
Sex (%)			0.0548
Female	51.31 (50.61,52.00)	53.11 (51.47,54.76)	
Male	48.69 (48.00,49.39)	46.89 (45.24,48.53)	
Race/ethnicity (%)			<0.0001
Mexican American	7.77 (6.65,9.06)	7.33 (6.04,8.86)	
Non-Hispanic White	69.05 (66.51,71.48)	72.25 (69.51,74.83)	
Non-Hispanic Black	10.91 (9.65,12.31)	10.43 (9.06,11.98)	
Other Hispanic	5.28 (4.49,6.19)	4.49 (3.71,5.42)	
Other Race	7.00 (6.35,7.71)	5.51 (4.71,6.44)	
Marry status (%)			<0.0001
Never married	19.23 (18.02,20.51)	9.78 (8.69,10.99)	
Married/Living with partner	64.31 (62.85,65.73)	66.08 (64.38,67.74)	
Divorced/Widowed/Separated	16.46 (15.71,17.24)	24.14 (22.96,25.37)	
Education status (%)			<0.0001
Less than high school	4.01 (3.60,4.46)	5.89 (5.12,6.78)	
High school	31.32 (29.72,32.96)	38.95 (37.06,40.87)	
More than high school	64.68 (62.85,66.47)	55.16 (53.02,57.28)	
Recreational activity (%)			<0.0001
No	40.91 (39.28,42.57)	58.82 (56.73,60.87)	
Yes	59.09 (57.43,60.72)	41.18 (39.13,43.27)	
Drinking status (%)			<0.0001
Never	10.16 (9.25,11.15)	12.12 (11.14,13.18)	
Now	78.27 (76.92,79.56)	67.58 (65.75,69.36)	
Former	11.57 (10.89,12.29)	20.30 (18.93,21.74)	
Smoking status (%)			<0.0001
Never	56.79 (55.46,58.10)	49.30 (47.64,50.97)	
Now	19.66 (18.66,20.69)	17.89 (16.70,19.13)	
Former	23.56 (22.58,24.56)	32.81 (31.22,34.44)	
Diabetes (%)			<0.0001
No	89.58 (88.97,90.17)	39.53 (37.78,41.31)	
Yes	10.42 (9.83,11.03)	60.47 (58.69,62.22)	
Hypertension (%)			<0.0001
No	71.93 (70.89,72.94)	27.66 (26.10,29.28)	
Yes	28.07 (27.06,29.11)	72.34 (70.72,73.90)	
CVD (%)			<0.0001
No	94.34 (93.92,94.73)	81.06 (79.84,82.21)	
Yes	5.66 (5.27,6.08)	18.94 (17.79,20.16)	
Depression (%)			<0.0001
No	93.18 (92.67,93.65)	89.74 (88.76,90.65)	
Yes	6.82 (6.35,7.33)	10.26 (9.35,11.24)	
Hyperlipidemia (%)			<0.0001
No	38.16 (36.99,39.35)	5.48 (4.72,6.36)	
Yes	61.84 (60.65,63.01)	94.52 (93.64,95.28)	
Stroke (%)			<0.0001
No	97.97 (97.74,98.17)	94.14 (93.43,94.78)	
Yes	2.03 (1.83,2.26)	5.86 (5.22,6.57)	
LC9	72.55 (72.11,72.99)	60.50 (60.01,60.98)	<0.0001
HEI-2015 diet score	40.13 (39.20,41.06)	36.62 (35.53,37.72)	<0.0001
Physical activity score	73.77 (72.77,74.77)	62.41 (60.91,63.91)	<0.0001
Nicotine exposure score	71.61 (70.54,72.68)	70.59 (69.40,71.79)	0.1583
Sleep health score	83.83 (83.27,84.38)	81.57 (80.73,82.41)	<0.0001
Health behavior score	67.29 (66.64,67.93)	62.71 (61.98,63.44)	<0.0001
BMI score	67.17 (66.36,67.98)	36.75 (35.76,37.74)	<0.0001
Blood lipids score	67.05 (66.34,67.77)	54.74 (53.69,55.79)	<0.0001
Blood glucose score	91.58 (91.19,91.97)	67.14 (66.10,68.19)	<0.0001
Blood pressure score	74.46 (73.80,75.11)	51.03 (50.14,51.91)	<0.0001
Health factors score	72.88 (72.42,73.34)	51.12 (50.57,51.67)	<0.0001
PHQ-9 score	92.26 (91.87,92.65)	89.19 (88.56,89.81)	<0.0001
Fasting glucose (mg/dl)	98.51 (98.02,99.00)	122.43 (120.85,124.02)	<0.0001
Sleep duration (h)	7.10 (7.07,7.13)	7.10 (7.05,7.15)	0.9992
HbA1c (%)	5.43 (5.42,5.44)	6.19 (6.15,6.23)	<0.0001
TC (mmol/L)	5.04 (5.01,5.06)	5.00 (4.95,5.04)	0.0611
TG (mmol/L)	1.10 (1.08,1.11)	2.04 (1.98,2.10)	<0.0001
HDL-C (mmol/L)	1.46 (1.45,1.47)	1.16 (1.14,1.17)	<0.0001
LDL-C (mmol/L)	2.96 (2.93,2.98)	2.95 (2.91,2.99)	0.7124
Income to poverty ratio	3.13 (3.06,3.20)	2.92 (2.84,3.00)	<0.0001
BMI (kg/m2)	27.73 (27.57,27.89)	33.69 (33.45,33.93)	<0.0001

Mean (95% CIs) are provided for continuous variables. Percentages are used to represent categorical variables. BMI, body mass index; CVD, Cardiovascular Disease; TG, Triglycerides; TC, Total cholesterol; HDL-C, High-density lipoprotein cholesterol; LDL-C, Low-density lipoprotein cholesterol; HbA1c, Glycosylated hemoglobin; MetS, Metabolic syndrome; HEI, Healthy Eating Index; PHQ-9, Patient Health Questionnaire‐9; LC9, Life’s Crucial 9.

### 3.2 The association between LC9 and MetS

**Table 2** examines the relationship between LC9 scores and MetS across three models. Model 1 serves as the unadjusted baseline, Model 2 incorporates adjustments for sex, age, and race, and Model 3 extends these adjustments to include household income to poverty ratio, education level, marital status, drinking status, and cardiovascular disease, as well as stroke. In Model 3, the OR (95% CI) for LC9 scores and MetS is 0.941 (0.939, 0.944), indicating that each one-unit increase in LC9 score is associated with a 5.9% decrease in the incidence of MetS. The OR (95% CI) for Health behavior score and MetS is 0.992 (0.990, 0.993), suggesting that each one-unit increase in Health behavior score is linked to a 0.8% decrease in the incidence of MetS. The OR (95% CI) for Health factors score and MetS is 0.948 (0.946, 0.950), indicating that each one-unit increase in Health factors score is associated with a 5.2% decrease in the incidence of MetS.

**Table 2:** The association between LC9 and MetS.

Exposure	Model 1 OR (95% CI)	P value	Model 2 OR (95% CI)	P value	Model 3 OR (95% CI)	P value
LC9	0.938 (0.936, 0.940)	<0.0001	0.942 (0.940, 0.944)	<0.0001	0.941 (0.939, 0.944)	<0.0001
LC9 quartile						
Q1	Reference		Reference		Reference	
Q2	0.528 (0.493, 0.566)	<0.0001	0.546 (0.509, 0.586)	<0.0001	0.563 (0.521, 0.608)	<0.0001
Q3	0.267 (0.248, 0.288)	<0.0001	0.293 (0.271, 0.316)	<0.0001	0.297 (0.273, 0.323)	<0.0001
Q4	0.071 (0.064, 0.079)	<0.0001	0.087 (0.078, 0.097)	<0.0001	0.088 (0.078, 0.099)	<0.0001
P for trend		<0.0001		<0.0001		<0.0001
Health behavior score	0.990 (0.989, 0.991)	<0.0001	0.989 (0.988, 0.990)	<0.0001	0.992 (0.990, 0.993)	<0.0001
Health factors score categorical						
Low (0–49)	Reference		Reference		Reference	
Moderate (50–79)	0.820 (0.771, 0.871)	<0.0001	0.814 (0.763, 0.868)	<0.0001	0.882 (0.822, 0.947)	0.0005
High (80–100)	0.592 (0.549, 0.638)	<0.0001	0.566 (0.523, 0.613)	<0.0001	0.648 (0.593, 0.707)	<0.0001
Health factors score	0.944 (0.943, 0.946)	<0.0001	0.947 (0.945, 0.949)	<0.0001	0.948 (0.946, 0.950)	<0.0001
Health factors score categorical						
Low (0–49)	Reference		Reference		Reference	
Moderate (50–79)	0.273 (0.257, 0.290)	<0.0001	0.286 (0.269, 0.305)	<0.0001	0.298 (0.279, 0.319)	<0.0001
High (80–100)	0.023 (0.020, 0.026)	<0.0001	0.030 (0.025, 0.034)	<0.0001	0.031 (0.027, 0.037)	<0.0001

Model 1: no adjustment.

Model 2: adjusted for sex, age and race.

Model 3: adjusted for sex, age, race, family income to poverty ratio, education level, marriage status, drinking status, CVD, and stroke.

MetS: Metabolic syndrome; LC9: Life’s Crucial 9; OR: odds ratios; CI: confidence intervals.

To assess the stability of these associations, LC9 scores were categorized into quartiles, while Health behavior score and Health factors score were divided into low, moderate, and high groups. The negative correlations were consistently observed across all groups, with more pronounced effects at higher scores. The negative correlations between LC9 and Health factors score with MetS were significantly stronger than the association between Health behavior score and MetS.

**Figure 2:**
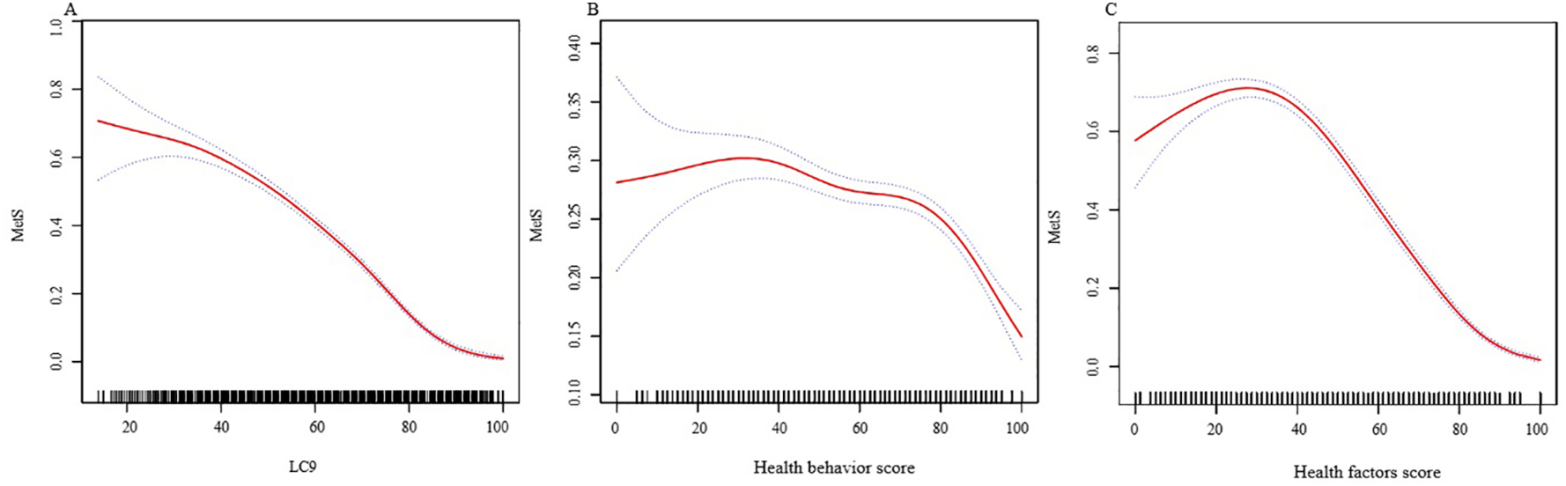
The associations between LC9 and MetS. **(A)** The associations between LC9 and MetS. **(B)** The associations between Health behavior score and MetS. **(C)** The associations between Health factors score and MetS. We adjusted for sex, age, race, family income to poverty ratio, education level, marriage status, drinking status, CVD, and stroke. MetS, Metabolic syndrome; LC9, Life’s Crucial 9; OR, odds ratios; CI, confidence intervals.

In further analyses, smooth curve fitting confirmed the curvilinear relationships between LC9, Health behavior score, and Health factors score with MetS, as depicted in **Figure 2**. **Table 3** employs a two-segment linear regression model to meticulously analyze the threshold effects of LC9, Health behavior score, and Health factors score on MetS. After accounting for all covariate factors, the inflection points for LC9, Health behavior score, and Health factors score were identified as 70.56, 86.25, and 46.25, respectively. Below the threshold of 70.56, the OR (95% CI) for LC9 and MetS is 0.96 (0.95, 0.96), and above the threshold, the OR (95% CI) is 0.88 (0.87, 0.89). This indicates that before the threshold, each one-unit increase in LC9 is associated with a 4% decrease in the incidence of MetS, and after the threshold, each one-unit increase in LC9 is associated with a 12% decrease in the incidence of MetS. For the association between Health behavior score and MetS, when Health behavior score is below 86.25, the OR (95% CI) is 0.99 (0.99, 1.00), and above 86.25, the OR (95% CI) is 0.95 (0.93, 0.96). For the association between Health factors score and MetS, when Health factors score is below 46.25, the OR (95% CI) is 1.00 (0.99, 1.00), and above 46.25, the OR (95% CI) is 0.93 (0.92, 0.93). The inflection point in the curvilinear relationship between Health factors score and MetS is significantly lower. Additionally, stronger correlations are evident above the threshold in all curvilinear associations.

**Table 3:** The threshold effects of LC9 on MetS were analyzed using a two-piecewise linear regression model.

Outcome:	Inflection point	OR (95% CI)	P value
LC9	< 70.56	0.96 (0.95, 0.96)	<0.0001
	> 70.56	0.88 (0.87, 0.89)	<0.0001
Health behavior score	<86.25	0.99 (0.99, 1.00)	<0.0001
	>86.25	0.95 (0.93, 0.96)	<0.0001
Health factors score	<46.25	1.00 (0.99, 1.00)	0.8608
	>46.25	0.93 (0.92, 0.93)	<0.0001
Sex			
Female	<72.78	0.96 (0.95, 0.96)	<0.0001
	>72.78	0.86 (0.85, 0.88)	<0.0001
Male	<68.33	0.96 (0.96, 0.97)	<0.0001
	>68.33	0.89 (0.88, 0.91)	<0.0001
Age			
<=60	<69.44	0.96 (0.95, 0.96)	<0.0001
	>69.44	0.87 (0.86, 0.88)	<0.0001
>60	<65.56	0.97 (0.97, 0.98)	<0.0001
	>65.56	0.91 (0.90, 0.92)	<0.0001
BMI			
<=30	<70.56	0.98 (0.97, 0.98)	<0.0001
	>70.56	0.90 (0.89, 0.91)	<0.0001
>30	<66.11	0.97 (0.97, 0.98)	<0.0001
	>66.11	0.93 (0.92, 0.94)	<0.0001

We adjusted for sex, age, race, family income to poverty ratio, education level, marriage status, drinking status, CVD, and stroke. BMI, body mass index; MetS, Metabolic syndrome; LC9, Life’s Crucial 9; OR, odds ratios; CI, confidence intervals.

**Figure 3:**
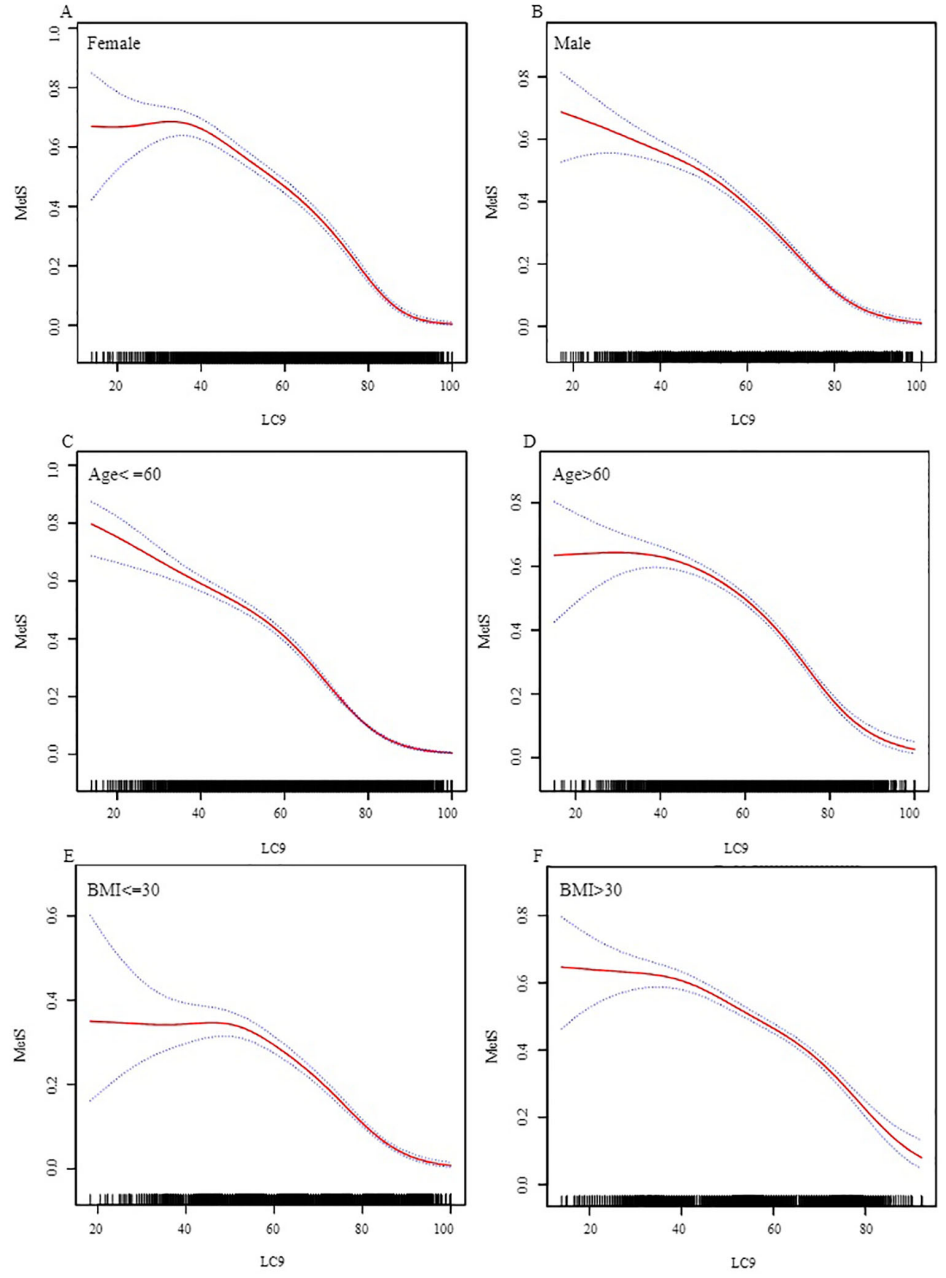
Stratified Analysis of the Association between LC9 and MetS. **(A)** Female. **(B)** Male. **(C)** Age<=60. **(D)** Age>60. **(E)** BMI<=30. **(F)** BMI>30.) We adjusted for sex, age, race, family income to poverty ratio, education level, marital status, drinking status, cardiovascular disease, and stroke, excluding the stratification variable itself. BMI, body mass index; MetS, Metabolic syndrome; LC9, Life’s Crucial 9; OR, odds ratios; CI, confidence intervals.

Stratified analyses by sex, age, and BMI indicate that LC9 exhibits a curvilinear relationship with MetS in all stratified analyses, consistent with the overall association (**Figure 3**). Sex-stratified analysis shows that the threshold is lower among male participants, yet the correlation is stronger after the inflection point among females. Age-stratified analysis indicates that participants over 60 have a lower threshold, but the correlation is stronger both before and after the inflection point among participants aged 60 and under. BMI-stratified analysis reveals that the threshold is lower in the group with a BMI over 30, with stronger correlations before the inflection point, while the correlation is stronger after the inflection point in the group with a BMI of 30 and under.

## 4 Discussion

Through the analysis of NHANES data, we found that after adjusting for covariates, there is a curvilinear relationship between LC9 and MetS. The inflection point is 70.56, and the relationship is negatively correlated both before and after the inflection point, with a more pronounced effect after the inflection point. While the associations between Health behavior score and MetS, as well as Health factors score and MetS, are also curvilinear and negatively correlated, there are significant differences. The inflection point for the curvilinear relationship between Health behavior score and MetS is 86.25, with ORs (95% CI) of 0.99 (0.99, 1.00) before the inflection point and 0.95 (0.93, 0.96) after the inflection point. For the curvilinear relationship between Health factors score and MetS, the inflection point is 46.25, with ORs (95% CI) of 1.00 (0.99, 1.00) before the inflection point and 0.93 (0.92, 0.93) after the inflection point. The differences are primarily reflected in the inflection points and the strength of the correlations: before the inflection point, Health behavior score is negatively correlated with MetS, while Health factors score is not, and after the inflection point, Health factors score shows a stronger negative correlation with MetS. This suggests that before the respective inflection points, improvements in Health behavior can reduce the incidence of MetS, while improvements in Health factors cannot. Although the correlation between Health behavior and MetS may not seem significant, the wide range of Health behavior score values means that good Health behavior can significantly reduce the incidence of MetS. The inflection point for the curvilinear relationship between Health factors score and MetS (46.25) is much lower than that for Health behavior score and MetS (86.25). Moreover, the correlation between Health factors score and MetS is more significant after the inflection point, indicating that improvements in Health factors can more effectively reduce the incidence of MetS after the inflection point. Therefore, we believe that both Health behavior score and Health factors score are important and complementary in the prevention and management of MetS.

In stratified analyses by sex, age, and BMI, the association between LC9 and MetS was found to be consistent with the overall pattern across all strata. In sex-stratified analysis, the inflection point for males (68.33) was lower than for females (72.78). The correlation was similar before the inflection point, but stronger for females after it. This suggests that males with LC9 scores between 68.33 and 72.78 are more likely to significantly reduce the incidence of MetS. When LC9 scores exceed 72.78, females are more likely to significantly reduce the incidence of MetS. Age-stratified analysis revealed that although the inflection point was slightly lower for participants over 60, the negative correlation between LC9 and MetS was more significant before and after the inflection point for those aged 60 and under. This indicates that younger individuals can achieve better prevention of MetS by increasing their LC9 scores. In BMI-stratified analysis, participants with a BMI over 30 had a lower inflection point and a more significant correlation before the inflection point. For participants with a BMI of 30 or less, the correlation between LC9 and MetS was more significant after the inflection point. This suggests that for obese participants, increasing LC9 scores before the respective inflection point is more effective in reducing the incidence of MetS. Conversely, for participants with a BMI of 30 or less, increasing LC9 scores after the inflection point is more effective in preventing MetS.

Currently, no studies have explored the association between LC9 and MetS. Our research marks the initial investigation into this relationship. However, existing evidence suggests that LE8 and depression are linked to MetS.

Reviewing the existing research, the findings are largely consistent, yet variations are also noted. Jia F et al. demonstrated a monotonically decreasing nonlinear dose-response relationship between the LE8 score and the predicted probability of MetS (26). Gou R et al. found that a higher LE8 score was associated with a lower incidence of MetS, with a stronger correlation observed for the health factors score (1). These findings align closely with our results. However, some studies have reported differing outcomes. Zhou DC et al. found that the LE8 score was negatively correlated with all-cause mortality and CVD mortality in individuals with MetS, with health behaviors playing a dominant role (27). Although this result may appear to contradict our findings, it actually strengthens our results. This study indicates that even in the presence of metabolic syndrome, characterized by a low Health factors score, healthy behaviors can effectively reduce the incidence of MetS. In prior research, depression has been significantly correlated with MetS. A cohort study conducted by Pimenta AM et al. revealed that the presence of depression was significantly associated with an increased risk of developing MetS (28). Additionally, a bidirectional two-sample Mendelian randomization study by Zhang M found a significant positive correlation between genetically predicted depression and the risk of MetS (29).

Depression is not only directly associated with the risk of MetS but also interacts with its constituent factors. Research indicates that diet quality is closely linked to depression (30). The sleep-wake cycle affects the onset and progression of depressive symptoms (31), and depression can also lead to sleep disorders (32). The relationship between smoking and depression may involve bidirectional interactions (33). Prospective observational studies have reported that each additional hour of physical activity per day or per week reduces the likelihood of developing depression (34). An elevated body fat percentage is closely associated with a higher prevalence of depression (35). Patients with Major Depressive Disorder experience systemic and localized impairments in glucose metabolism throughout the disease course (36). High levels of HDL-C are negatively correlated with depression, while TG levels is positively correlated with depression (13). A prospective cohort study shows that depressive symptoms are a risk factor for new-onset home hypertension, particularly for the occurrence of nocturnal hypertension in individuals with normal home blood pressure (37).

In conclusion, the effects of LE8 and depression on MetS are likely not unidirectional but involve a complex bidirectional relationship with intersecting influences among the various factors.

Our research utilizes data from the NHANES database, renowned for its meticulous data collection and extensive sample sizes, which underpins the validity and dependability of our findings. Through the application of stratified analysis, we have investigated the link between LC9 and MetS, as well as how this relationship differs across various demographic segments. Nevertheless, our study is subject to certain inherent constraints. Primarily, as a cross-sectional observational study, it cannot establish a causal link between MetS and LC9, thus emphasizing the necessity for longitudinal research to ascertain causality and the chronological order of events. Additionally, despite adjusting for a multitude of covariates, there may still be unaccounted confounding variables influencing the association between LC9 and MetS, such as personal lifestyle choices and genetic factors. Furthermore, disparities in socioeconomic standing and access to healthcare services could potentially influence the results of our study. The scope of the study and the regional boundaries of the data imply that additional clinical investigations are warranted in diverse countries and regions to delve into the mechanistic connections between LC9 and MetS.

## 5 Conclusions

Our in-depth analysis of the data revealed curvilinear relationships between LC9 scores, health behavior scores, health factors scores, and MetS. We identified their respective inflection points and calculated the correlations before and after these points. This allows us to better understand the impact of changes in LC9 scores, health behavior scores, and health factors scores on the incidence of MetS and the existing differences. We conducted stratified analyses for different populations to reveal the differences in the association between LC9 scores and MetS across various groups. We believe that studying the relationship between LC9 and MetS is crucial for the prevention of MetS. Our study provides a more comprehensive perspective on the prevention and management of MetS.

## Data Availability

Publicly available datasets were analyzed in this study. This data can be found here: wwwn.cdc.gov/nchs/nhanes/Default.aspx.
